# Neurocognitive Predictors of ADHD Outcome: a 6-Year Follow-up Study

**DOI:** 10.1007/s10802-016-0175-3

**Published:** 2016-07-09

**Authors:** Marloes van Lieshout, Marjolein Luman, Jos W. R. Twisk, Stephen V. Faraone, Dirk J. Heslenfeld, Catharina A. Hartman, Pieter J. Hoekstra, Barbara Franke, Jan K. Buitelaar, Nanda N. J. Rommelse, Jaap Oosterlaan

**Affiliations:** 1 Department of Clinical Neuropsychology, VU University Amsterdam, Van der Boechorststraat 1, 1081 BT, Amsterdam, The Netherlands; 2 Department of Health Sciences, VU University Amsterdam, Amsterdam, The Netherlands; 3 Department of Epidemiology and Biostatistics, VU University Medical Center, Amsterdam, The Netherlands; 4 Departments of Psychiatry and of Neuroscience and Physiology, SUNY Upstate Medical University, Syracuse, NY USA; 5 KG Jebsen Centre for Research for Neuropsychiatric Disorders, University of Bergen, Bergen, Norway; 6 Department of Psychiatry, University Medical Center Groningen, University of Groningen, Groningen, The Netherlands; 7 Department of Psychiatry, Donders Institute for Brain, Cognition and Behaviour, Radboud University Medical Center, Nijmegen, The Netherlands; 8 Department of Human Genetics, Radboud University Medical Center, Nijmegen, The Netherlands; 9 Department of Cognitive Neuroscience, Donders Institute for Brain, Cognition and Behaviour, Radboud University Medical Center, Nijmegen, The Netherlands; 10 Karakter Child and Adolescent Psychiatry University Center, Nijmegen, The Netherlands

**Keywords:** ADHD, Outcome, Neurocognitive functioning, Prediction

## Abstract

Although a broad array of neurocognitive dysfunctions are associated with ADHD, it is unknown whether these dysfunctions play a role in the course of ADHD symptoms. The present longitudinal study investigated whether neurocognitive functions assessed at study-entry (mean age = 11.5 years, *SD* = 2.7) predicted ADHD symptom severity and overall functioning 6 years later (mean age = 17.4 years, 82.6 % = male) in a carefully phenotyped large sample of 226 Caucasian participants from 182 families diagnosed with ADHD-combined type. Outcome measures were dimensional measures of ADHD symptom severity and the Kiddie-Global Assessment Scale (K-GAS) for overall functioning. Predictors were derived from component scores for 8 domains of neurocognitive functioning: working memory, motor inhibition, cognitive inhibition, reaction time variability, timing, information processing speed, motor control, intelligence. Effects of age, gender, and pharmacological treatment were considered. Results showed that better working memory predicted lower ADHD symptom severity (*R* ^*2*^ = 3.0 %), and less reaction time variability predicted better overall functioning (higher K-GAS-score, *R* ^*2*^ = 5.6 %). Predictors were still significant with baseline behavior included in the models. The role of neurocognitive functioning in the long term outcome of ADHD behavior is discussed.

**Electronic supplementary material:**

The online version of this article (doi:10.1007/s10802-016-0175-3) contains supplementary material, which is available to authorized users.

Attention-Deficit/Hyperactivity Disorder (ADHD) is a common developmental disorder, with impairing and highly persistent symptoms of inattention and/or hyperactivity/impulsivity (Biederman et al. 2011). Impairment exists in several domains of functioning, including academic, social, and occupation functioning (Barkley et al. 2006). Patients with persistent ADHD experience chronic problems in adult life compared to those with remitted ADHD, such as higher rates of substance use disorders (Klein et al. 2012) and other psychiatric comorbidities (Barbaresi et al. 2013). It is important to elucidate factors involved in the course of ADHD, since ‘baseline’ predictors that would allow prediction of the course of ADHD in terms of ADHD symptom severity and overall functioning could be used to inform parents and children. Such information may also guide further study into treatment; for example investigating whether neurocognitive training of a particular predictive function might enhance prognosis.

Neurocognitive functioning may act as predictor of symptom severity and overall functioning of ADHD, as neurocognitive dysfunction is a key aspect of the disorder (Willcutt et al. 2008) and is at the heart of several models of ADHD (Barkley 1997; Sergeant 2000; Sonuga-Barke et al. 2010). The relevance of neurocognitive functioning for the course of ADHD has been emphasized by Halperin and Schulz (2006). According to their model, higher-order mental functions (so-called cognitive control functions as inhibition and working memory) may contribute to better outcome.

To evaluate the hypothesis that neurocognitive functioning may act as a predictor for ADHD outcome, we recently reviewed existing literature. Some of the studies showed positive associations between concurrently assessed neurocognitive performance and ADHD outcome in adolescents (Barkley and Fischer 2011; Coghill et al. 2014; Halperin et al. 2008). However, as baseline measures of neurocognitive performance were not available in most of these studies, it remains unknown whether the associations were apparent earlier in childhood yet. On the basis of six studies that investigated the predictive value of early assessed neurocognitive functions, we concluded that ADHD symptom remission was not predicted by neurocognitive functioning (van Lieshout et al. 2013). This conclusion was largely supported by more recent studies. One study showed that only one out of nine neurocognitive functions (attentional set-shifting) predicted greater decrease in ADHD symptoms 10 years later (Coghill et al. 2014). In addition, recent work has shown that neurocognitive functions in childhood were not related to ADHD outcome in adolescence (McAuley et al. 2014), and that neurocognitive functions in 3-to-4 year olds were not related to changes in ADHD severity 4.5 years later (Rajendran et al. 2013a, b). One study showed that an aggregated measure of neurocognitive functioning was related to ADHD severity 1 year later in children between 5 and 7 years but not in children between 4 and 5 years: better functioning predicted lower ADHD severity (Rajendran et al. 2013a, b). Finally, in one study that assessed ADHD symptoms, two out of four neurocognitive measures (working memory and reaction time variability) in preschool predicted future symptoms of inattention (Sjöwall et al. 2015). Taken together, there is only little evidence to predict ADHD outcomes based on early neurocognitive functions.

However, these findings may be related to limitations of the available literature. The first limitation is related to the type of outcome measure chosen. Available studies have focused mainly on dichotomous outcomes (diagnosis yes/no), rather than on more sensitive continuous measures of symptom severity (Lahey and Willcutt 2010; Willcutt et al. 2012). Also, some studies exclusively focused on ADHD core symptoms and did not assess accompanying levels of impairment. Outcome as measured in terms of the level of overall functioning may clinically be more relevant. The second limitation relates to the type of neurocognitive assessment used. Only few studies were conducted on key neurocognitive functions associated with ADHD, such as cognitive control, temporal processing and reward processing (van Lieshout et al. 2013). Also, most of the longitudinal studies did not consider a full set of neurocognitive predictors together, which is important as predictors may overlap. Focusing on one domain also narrows clinical applicability. In addition, not many studies investigated whether early neurocognitive functions predict outcomes over and above ADHD behavior, which is of importance to rule out the possibility that neurocognitive functions act simply as a proxy for ADHD severity. Fifth, most studies have not investigated developmental and gender effects. Regarding developmental effects, it is thought that development of an individual is ongoing from childhood into adulthood with a sharp transition period in adolescence (Geier 2013). Also, neurocognitive development is likely to be non-linear (Vaughn et al. 2011). Therefore, it is important to consider moderating effects of age when investigating outcomes over time. Previous studies so far investigated a narrow age range or did not specifically investigate possible moderating effects of age. For example, only in very young children (3 to 6 years), neurocognitive functioning predicted ADHD status or severity several years later (Rajendran et al. 2013a, b; van Lieshout et al. 2013). Also, it is possible that gender might impact on results, given differences in brain structure and function in healthy controls (Bell et al. 2006), and that prevalence rates of ADHD are higher in males than in females (Willcutt 2012). Finally, few studies took effects of pharmacological treatment into account. This may be of importance, as pharmacological treatment may impact on outcomes; for example, in a meta-analysis including 23 studies, it was found that both amphetamine and methylphenidate products were efficacious in treating ADHD symptoms (Faraone and Buitelaar 2010).

The current study addressed the abovementioned issues by employing a dimensional approach to investigate the predictive value of neurocognitive functioning for (1) ADHD symptom severity and (2) overall functioning, using a longitudinal design with a 6-year follow-up of children with combined-type ADHD (ADHD/C). We investigated children in the full range of childhood age, with careful consideration of age-dependent effects. As ADHD is associated with heterogeneity in neurocognitive deficits (Nigg et al. 2005), we assessed a broad array of neurocognitive functions to capture as much as possible this heterogeneity. We used measures of cognitive control (motor inhibition, cognitive inhibition, working memory) and temporal processing (variability in responding, timing), as well as other functions that show impairments in individuals with ADHD (basic information processing speed, motor control, and intellectual functioning). In addition, neurocognitive performance may differ between subtypes (Dovis et al. 2015). Therefore, including only participants with ADHD/C and not participants with the inattentive or hyperactive/impulsive subtype, might increase homogeneity in neurocognitive functioning as well. The predictive value of neurocognitive functions for ADHD symptom severity and overall functioning was studied taking into account baseline symptom severity and impairment as well, respectively. Age, gender, pharmacological treatment and study site were additionally considered as confounding variables. Taking into account limitations of earlier studies, we tested the hypotheses that better early neurocognitive functioning would be associated with lower symptom severity and better overall functioning at follow-up. As the available literature did not allow us to form specific hypotheses, we expected similar results for ADHD symptom severity and overall functioning (i.e., better neurocognitive functioning related to better outcomes: lower symptom severity and better overall functioning), since overall functioning is highly dependent on the expression and consequences of the primary symptoms (Caci et al. 2015).

## Method

### Participants

Participants (*N* = 459) with a DSM-IV-TR diagnosis of ADHD/C aged 5–19 years were recruited from outpatient clinics and via advertisements between 2003 and 2006 in the Dutch part of the International Multicenter ADHD Genetics (IMAGE) study. Six years later, subjects were invited for a comprehensive follow-up assessment as part of the NeuroIMAGE study (von Rhein et al. 2015). The period between baseline and follow-up assessment was on average 6.0 years (*SD* = 0.7) and 347 participants (75.6 %) were retained successfully. Of these 347 participants, 226 participants participated in the neuropsychological assessment during the IMAGE study (baseline measurement) and were included in the current study. Results of attrition analyses are described in the Results section.

Selection and diagnostic procedures at baseline have been detailed previously (Müller et al. 2011a, 2011b). Briefly, inclusion criteria for entry at baseline were an age of 5–19 years, Caucasian descent, IQ ≥ 70, no diagnosis of autism, epilepsy, general learning difficulties, brain disorders, and known genetic disorders, and having at least one sibling (regardless of ADHD status). The parent and teacher Conners’ long version (Conners et al. 1998) and Strengths and Difficulties Questionnaire (SDQ; Goodman 1997) were used to screen participants: *T*-scores ≥63 on the Conners’ ADHD subscales L (DSM-IV Inattentive symptoms), M (DSM-IV Hyperactive/impulsive symptoms), and N (DSM-IV Total symptoms), and scores ≥90th percentile on the SDQ Hyperactivity subscale were considered clinical. Participants obtaining clinical scores on any of these subscales were administered the Parental Account of Children’s Symptoms (PACS), a semi-structured, standardized, investigator-based interview with the parents as informants (Taylor 1986). See Rommelse et al. (2007a); b; c) for the algorithm used to derive each of the 18 ADHD symptoms as defined by the DSM-IV-TR (American Psychiatric Association 2000). The 226 participants included in the current study with ADHD/C at baseline came from 182 different families. Their mean age at baseline was 11.5 years (*SD* = 2.7), and 17.4 years (*SD* = 2.7) at follow-up, and 82.6 % was male. Regarding medication use at follow-up, 88.5 % of all participants used stimulants, 11.9 % of all participants used atomoxetine, 4.9 % of all participants used antidepressants, and 1.3 % of all participants used tranquillizers (e.g., benzodiazepines, anxiolytics).

#### Outcome Measures

At follow-up, ADHD total symptom severity as well as inattentive and hyperactive/impulsive symptom severity were assessed with the Conners’ Parent Rating Scale–Revised: Long version (CPRS-R:L; Conners et al. 1998) scales N (DSM-IV Total symptoms; Cronbach’s α = 0.93), L (DSM-IV Inattentive symptoms; Cronbach’s α = 0.90) and M (DSM-IV Hyperactive/impulsive symptoms; Cronbach’s α = 0.87), respectively. Scores on the Conners’ ADHD subscales represent combined measures of the number (maximum 18) and severity (range 0–3) of symptoms, with scores ranging between 0 and 54 (maximum number of symptoms; 18, with maximum severity; 3), or between 0 and 27 (maximum number of symptoms within one symptoms axis; 9, with maximum severity; 3), respectively. Raw scores were used.

The Global Assessment Scale-score (K-GAS) of the Dutch version of the Schedule for Affective Disorders and Schizophrenia for School-Age Children - Present and Lifetime Version (K-SADS; Kaufman et al. 1997) administered at follow-up to both the parent and the child ≥12 years separately, was used to measure overall functioning. This measure is a time-efficient and clinically relevant measure of overall functioning. After finishing the K-SADS interview, the interviewer rated psychological, academic and social functioning. This resulted in an overall measure of the current level of functioning ranging between 1 (worst possible level of functioning) and 9 (best possible level of functioning; Schorre and Vandvik 2004).

For both the K-SADS and the PACS, interviewers of the participating centers underwent comprehensive training by a team under the supervision of E. Taylor at the London Institute of Psychiatry (PACS) or JB at the Donders Institute for Brain, Cognition and Behavior, Radboud University Medical Centre, Nijmegen (K-SADS). Trained interviewers used the same training and supervision procedures for additional interviewers at the participating centers. Inter-rater agreement for the PACS was 0.88 (range 0.71–1.00; Müller et al. 2011a) and for the K-SADS 0.94 (ADHD), 0.89 (ODD), and 0.95 (CD; von Rhein et al. 2015). The interviewers were trained clinicians (child psychiatrists, psychologists) or trained researchers.

#### Predictor Variables

We investigated eight domains of neurocognitive functioning measured at baseline: working memory, motor inhibition (the ability to stop a prepotent response), cognitive inhibition (the ability to flexibly shift between two response options), reaction time variability, timing, information processing speed, motor control, and intelligence. Domains were chosen to include a broad array of neurocognitive tasks (see Table 1) that are known to be sensitive to detect differences between children with ADHD and control children (Rommelse et al. 2008a, b; c; Rommelse et al. 2008a, b; c), and that yield enough variance in order to be useful as predictors of differences in symptom severity and overall functioning (Nigg et al. 2005). For each neurocognitive domain, we performed a principal component analysis (PCA) to optimize the number of predictor variables and reduce error variance; a total of eight principal component analyses thus were performed. First, we selected the most widely used and theoretically valid independent measures for each domain, thereby including at least two measures in each PCA with the exception of the domain of intelligence, for which only one measure (based on four subtests) was available. When two measures from one task within a domain correlated >0.85, one of the measures was excluded from the PCA to prevent clustering of highly correlated variables.

**Table 1:** Description of Instruments

Task	Measure	Description	References
WISC/WAIS-III digit span	- Maximum Span Forwards - Maximum Span Backwards	An auditory task to measure the accuracy of verbal working memory; a sequence of numbers was announced, and should be replicated forwards (or backwards in the backwards condition), with increasing length.	Wechsler 2000/Wechsler 2002
ANT visuo-spatial sequencing task	- Total number of identified targets in correct order	A computerized task to measure the accuracy of visuo-spatial working memory; a sequence of circles in a 3 × 3 grid should be replicated in the correct order by pointing to them, with increasing length.	Rommelse et al. 2008a, b; c de Sonneville 1999
Stop task	- RT on go-trials (ms) - SDRT on go-trials (ms), corrected for MRT. - Stop Signal Reaction Time (ms) - Percentage commission errors	A computerized task to measure the speed and accuracy of inhibition of an ongoing response; go-trials required an accurate response to an external cue, with two choices (left or right); stop-trials required no response to an external cue.	Rommelse et al. 2008a, b; c Logan and Cowan 1984
ANT shifting attentional set	- RT Block 1 (ms) - SDRT Block 1, corrected for MRT	Block 1: A computerized task to measure the speed and variability of motor output in response to an external cue, with two choices (left or right).	Rommelse et al. 2007a, b; c de Sonneville 1999
	- RT Block 2 (ms), corrected for MRT Block 1 - Percentage errors, corrected for errors Block 1	Block 2: As in block 1, but in block 2 an *incompatible* response was required (pressing a button in the direction opposite to which the stimulus moved).	
ANT baseline speed	- RT (ms) - SDRT, corrected for MRT.	A computerized task to measure the speed and variability of motor output in response to an external cue (simple reaction time task)	Rommelse et al. 2008a, b; c de Sonneville 1999
Motor timing task	- SDRT, corrected for MRT (ms) - Mean absolute deviation (ms)	A computerized task to measure the accuracy and variability of motor timing, requiring a response on a button when a subject thought a 1-s interval had elapsed after a tone.	Rommelse et al. 2008a, b, c, van Meel et al. 2005
Time test	- Percentage of deviation visual modality (mean of three highest intervals) - Percentage of deviation auditory modality (mean of tree highest intervals)	A computerized task to measure the precision of the reproduction of five time intervals (4, 8, 12, 16, 20s); A light bulb (visual modality) or a tone (auditory modality) was presented for a specific interval length, which had to be reproduced thereafter.	Rommelse et al. 2007a, b; c Barkley 1998
ANT pursuit	- Absolute deviation left hand	A computerized task to measure the precision of motor control; a randomly moving target should be followed as precisely as possible by moving a mouse cursor.	Rommelse et al. 2007a, b; c de Sonneville 1999
ANT tracking	- Absolute deviation left hand	A computerized task to measure the precision of motor control; an invisible midline should be traced with a mouse cursor as quickly and precisely as possible, between an inner and an outer circle.	Rommelse et al. 2007a, b; c de Sonneville 1999
WISC/WAIS-III vocabulary, similarities, block design, picture completion	- Estimated total IQ	Four subtests of the WISC/WAIS were used to estimate full-scale IQ.	Wechsler 2000/Wechsler 2002

*ANT* Amsterdamse Neuropsychologische Taken, *MRT* Mean reaction time, *RT* Reaction time, *SDRT* Standard deviation of reaction time, *WAIS* Wechsler Adult Intelligence Scale, *WISC* Wechsler Intelligence Scale for Children

Table 2 provides a description of the neurocognitive domains and corresponding measures that were finally included in our principal component analyses, with descriptive information and the principal component score per measure. For working memory, we initially included four measures. Of these four measures, ‘number of identified targets’ (NIT) and ‘number of identified targets in the correct order’ (NITco) correlated >0.85 with each other. Therefore, we decided to discard NIT from the PCA, because NITco not only captures increasing length (load), but also captures correct order, hence offering a more valid measure of working memory (Baddeley 2012). For motor control, we initially included the mean absolute deviation and the standard deviation of the mean distance of two motor control measures (Pursuit and Tracking). However, both measures of the standard deviation correlated >0.85 with the mean absolute deviation on the corresponding task. As the mean absolute deviation on the task is the most widely used measure of motor control, we decided to discard the standard deviations of both tasks from the PCA. The PCA was conducted using a correlation matrix, calculated on standardized data. Results showed one component with an Eigenvalue >1 (cut-off value) for each domain. Principal components were rescaled so that higher scores represent better performance. For reaction time variability, smaller variability was interpreted as better performance. See Table 3 for a description of group means and standard deviations of the predictor and outcome variables. Supplemental Table 1(available online) provides correlations between baseline/follow-up behavioral variables (symptom severity, overall functioning and impairment) and neurocognitive predictors.

**Table 2:** Description of Principal Components

Neurocognitive domain	Task	Measure	*M*	*SD*	Range (min-max)	Component scores
Working memory	ANT visuo-spatial sequencing task	Total number of identified targets in correct order	87.81	12.20	39.0–105.0	.42
	WISC/WAIS-III digit span	Maximum span forwards	5.21	1.10	3.0–9.0	.41
		Maximum span backwards	3.81	1.12	2.0–7.0	.45
Motor inhibition	Stop task	SSRT	293.87	90.60	100.0–608.1	.54
		Percentage commission errors	3.64	3.54	0.0–15.1	.54
Cognitive inhibition	ANT shifting attentional set, block 2	Reaction time (corrected^b^) (ms)	230.84	174.11	−243.7-871.4	.63
		Percentage errors (corrected^b^)	8.86	12.55	−15.0-50.0	.63
Reaction time variability	ANT baseline speed	SDRT (corrected^a^, ms)	0.40	0.24	0.14–1.2	.24
	ANT shifting attentional set, block 1	SDRT (corrected^a^, ms)	0.37	0.18	0.10–1.0	.41
	Stop task	SDRT go trials (corrected^a^, ms)	0.23	0.04	0.13–0.35	.41
	Motor timing	SDRT (corrected^a^, ms)	0.28	0.09	0.07–0.58	.43
Timing	Motor timing	Mean absolute deviation (ms)	273.50	146.90	49.9–741.7	.38
	Time test, visual modality	Percentage of deviation	21.34	13.76	2.4–63.6	.39
	Time test auditory modality	Percentage of deviation	24.19	14.62	4.1–69.3	.39
Information processing speed	ANT baseline speed	Reaction time (ms)	360.11	81.38	224.0–638.0	.42
	ANT shifting attentional set, block 1	Reaction time (ms)	269.08	920.50	269.1–920.5	.41
	Stop task	Reaction time go trials (ms)	591.41	114.48	357.8–1051.1	.36
Motor control	ANT pursuit	Absolute deviation left hand	5.90	2.98	2.0–15.0	.58
	ANT tracking	Absolute deviation left hand	2.85	1.83	0.5–8.4	.58
Intelligence	WISC/WAIS-III vocabulary, similarities, block design, picture completion	Total IQ	99.46	11.68	70.0–133.0	-

*ANT* Amsterdamse Neuropsychologische Taken, *PCA* Principal Component Analysis, *SDRT* Standard Deviation of Reaction Time, *SSRT* Stop Signal Reaction Time, *WISC* Wechsler Intelligence Scale Children, *WAIS* Wechsler Adult Intelligence Scale-third edition

^a^Corrected for mean reaction time

^b^Corrected for reaction time or errors in block 1

**Table 3:** Descriptives of Predictor and Outcome Variables

	*M*	*min*	*max*	*SD*
Baseline Variables				
CPRS-R:L Inattentive symptom severity (scale L)	18.55	2.00	27.00	5.07
CPRS-R:L Hyperactive/impulsive symptom severity (scale M)	16.73	2.00	27.00	5.39
CPRS-R:L Total symptom severity (scale N)	35.29	6.00	52.00	8.99
SDQ Impairment (parent)	11.98	0.00	21.0	3.91
Follow-up Variables				
CPRS-R:L Inattentive symptom severity (scale L)	14.09	0.00	27.00	6.38
CPRS-R:L Hyperactive/impulsive symptom severity (scale M)	9.26	0.00	27.00	5.80
CPRS-R:L Total symptom severity (scale N)	23.32	0.00	52.00	11.01
K-GAS-Score	6.49	2.00	9.00	1.08
Follow-up Interval	5.85	4.40	7.68	0.65
Pharmacological treatment (cumulative intake of stimulants)	118.27	0.00	477.44	111.88

Scores of neurocognitive measures are component scores. *ADHD* Attention-Deficit/Hyperactivity Disorder *CPRS* Conners’ Parent Rating Scale-Revised: Long version, *K-GAS* Kiddie-Global Assessment Score, *SDQ* Strengths and Difficulties Questionnaire, *PC* Principal Component

#### Covariates

As there is a strong relationship between baseline behavior and behavior at follow-up, models predicting symptom severity were calculated with and without baseline ADHD symptom severity (CPRS-R:L scale N: DSM-IV Total symptoms; Cronbach’s α = 0.85). Likewise, baseline impairment was included when predicting overall functioning. Impairment at baseline was measured by the impairment scale of the Strengths and Difficulties Questionnaires (SDQ; Cronbach’s α = 0.75; Goodman 1997), reported by parents (range 0–21). When follow-up interval was significantly related to current symptom severity, follow-up interval was included as a covariate in further analyses. Follow-up interval was defined as the time between baseline and follow-up measurement (in years). The same procedure was followed when overall functioning was predicted. Age and age^2^ (assessed at baseline), gender, pharmacological treatment until follow-up and study site (Amsterdam or Nijmegen) were added as covariates to the final models. Pharmacological treatment was collected in terms of the cumulative intake of psychostimulants (mean daily dose multiplied with treatment duration corrected for age) from age of onset until the follow-up assessment using information from pharmacy records supplemented with information from parent questionnaires. See van Lieshout et al. (2016) for further details.

### Procedure

Testing at baseline and follow-up took place at the VU University Amsterdam, or at the Donders Institute for Brain, Cognition and Behaviour, Radboud University in Nijmegen, the Netherlands. Participants were 48 h off medication before both baseline and follow-up assessments. All ratings of behavioral functioning pertained the participant’s functioning off medication. Families were financially compensated for participation. Informed consent was signed by all participants at both measurements, and parents signed for all children in their family as well. The study was approved by the national and local ethics committees.

### Statistical Analysis

In the sample of 226 children, the Stop task was not administered to 9.7 % of participants due to technical problems. Between 0.4–3.1 % of data were missing for other neurocognitive predictors. Missing value analysis (Expectation Maximization with 25 iterations) for Stop Task data was performed only for participants with at least nine out of ten neurocognitive tasks available (*n* = 22). Percentage of missing data at follow-up was 2.2 % for ADHD symptom severity measures, and 3.1 % for overall functioning measures. All outcome variables had a normal distribution with values of skewness and kurtosis within the range of −1 to +1, except for the K-GAS score (kurtosis = −1.20). K-GAS-scores were normalized by applying a Van der Waerden transformation.

To optimally correct for the familial dependency in our data, Generalized Estimating Equation analyses (GEE) were used with an exchangeable correlation structure. An optimal set of predictors for (1) symptom severity and (2) overall functioning was derived by performing a backward selection procedure (variables deleted when *p* > 0.05), until an optimal final model was composed. The mean correlation between predictors was 0.36 (0.03 ≤ *r* ≤ 0.70), indicating no collinearity. Analyses predicting symptom severity or impairment used three steps: (1) Models predicting symptom severity or impairment were calculated with the neurocognitive predictors only (model 1). (2) To investigate the additional predictive value of neurocognitive functioning over and above baseline behavior or baseline impairment, models were calculated with the neurocognitive predictors together with baseline symptom severity or baseline impairment (model 2). (3) Then, models were calculated with baseline symptom severity or baseline impairment only, for comparison (model 3). We described differences between the models (1) and (2), by evaluating the difference in *R* ^*2*^.

#### Additional Analyses

To investigate age effects, both age and quadratic effects of age were examined. Interactions between age and significant predictors of outcome were added to the final models with neurocognitive functioning (model 1). When an interaction-effect with age or age^2^ was significant, the finding was further explored by testing the final model in subsamples subdivided based on age at baseline (<12 years and 12 > = years). The same procedure was followed for gender. The final model of ADHD symptom severity (model 1) was reran using both inattentive and hyperactive/impulsive symptoms as outcome measures (including baseline symptom severity), to explore whether the model was applicable to both symptom axes.

#### Sensitivity Analyses

As the reliability and validity of the CPRS-R:L is only established for children under 18 years of age, we checked whether results of the final model for ADHD symptom severity (model 1) were robust when tested in children younger than 18 years. Beside possible moderating effects of age and gender, we tested possible effects of confounders (age, age^2^, gender, pharmacological treatment, study site) on the final models.

## Results

### Attrition Analyses

Attrition was investigated by comparing participants successfully followed up (i.e., included in our analyses, *N* = 226) with participants lost to follow-up from the total sample on 25 variables available at baseline (age, gender, ADHD symptoms, neurocognitive measures). Participants who were lost to follow-up had higher SD (corrected for MRT) on the motor timing task (*p* < 0.001) and had more commission errors on the Stop task (*p* = 0.036)*.* No other significant group differences were found (0.070 < *p* < 0.983).

### Prediction of ADHD Symptom Severity

Follow-up interval was not related to current ADHD symptom severity (*p* = 0.675). Table 4 shows the final prediction model. Better working memory predicted lower ADHD symptom severity, explaining 3.0 % of variance. When taking baseline symptom severity into account, better working memory still predicted lower ADHD symptom severity, together explaining 11.7 % of variance. For comparison, baseline symptom severity alone explained 10.0 % of variance.

**Table 4:** Final Prediction Models for Current ADHD Symptom Severity in Children with ADHD/C

	*b*	*SE*	*p*
Model 1			
Working memory	−1.34	0.45	0.003
	*R* ^*2*^ = 3.00 %		
Model 2			
Working memory	−1.05	0.51	0.041
CPRS-R:L symptom severity	0.43	0.06	<0.001
	*R* ^*2*^ = 11.71 %		

*b* is the unstandardized coefficient

*ADHD* Attention-deficit/hyperactivity disorder, *CPRS-R:L* Conners’ Parent Rating Scale-Revised: Long version

### Prediction of Overall Functioning

Follow-up interval was related to the K-GAS-score (*b* = −0.29, *p* < 0.001) and therefore included as a covariate in all further analyses. Table 5 shows the final prediction model. Higher reaction time variability predicted lower K-GAS-scores, explaining 5.6 % of variance. When taking baseline parent-reported impairment into account, higher reaction time variability still predicted lower K-GAS-scores, together explaining 8.6 % of variance. For comparison, baseline parent-reported impairment alone explained 7.1 % of variance.

**Table 5:** Final Prediction Models for Current Overall Functioning in Children with ADHD/C

	*b*	*SE*	*p*
Model 1^a^			
Reaction time variability	0.11	0.05	.018
	*R* ^*2*^ = 5.60 %		
Model 2^a^			
Reaction time variability	0.10	0.04	.030
SDQ impairment parent	−0.04	0.01	.003
	*R* ^*2*^ = 8.62 %		

*b* is the unstandardized coefficient

*ADHD* Attention-deficit/hyperactivity disorder, *SDQ* Strengths and Difficulties Questionnaire

^a^Models are adjusted for follow-up interval

### Additional Analyses

#### Age and Gender Effects

Significant neurocognitive predictors for both ADHD symptom severity (working memory) and overall functioning (reaction time variability) did not significantly interact with age (*b* = 0.02, *p* = 0.882/*b* = 0.01, *p* = 0.499 respectively), age^2^ (*b* < 0.001, *p* = 0.978/*b* < 0.001, *p* = 0.621 respectively), or gender (*b* = −1.09, *p* = 0.411/ *b* = −0.13, *p* = 0.231, respectively).

#### Effects of Predictors on Inattention and Hyperactivity/Impulsivity

Working memory (model 1) was not a significant predictor for ADHD inattention symptom severity (*p* = 0.174), but was a significant predictor for current ADHD hyperactivity/impulsivity symptom severity; better working memory pre-dicted lower hyperactivity/impulsivity symptom severity (*p* = 0.001). *R* ^*2*^ for the final model of hyperactivity/impulsivity was 3.91 %.

### Sensitivity Analyses

#### Sample < 18 years

Predictors relevant in the final model (model 1) for current ADHD symptom severity were significant with similar relationships when tested in a subsample of children younger than 18 years (*b* subsample = −1.43 /β = −0.15, *p* = 0*.*040, *R* ^*2*^ = 1.6 %, versus *b* full sample = −1.34 / β = −0.17; *p* = 0*.*003, *R* ^*2*^ = 3.0 %). Taking into account that the sample size of this subsample was substantially smaller, these findings suggest that the results also hold when the age group was excluded for which the CPRS-R:L was not validated.

#### Covariates

Findings for current ADHD symptom severity as well as for overall functioning replicated when age or age^2^, gender, pharmacological treatment, or study site were added as covariates to the final models (model 1). Covariates were not significant in the final models for ADHD symptom severity and overall functioning (0.068 < *p* < 0.830).

## Discussion

The current large prospective study investigated whether a broad array of well-defined neurocognitive measures predicted dimensional outcome measures of ADHD, 6 years later, in children and adolescents with ADHD-combined type. In summary, better working memory predicted lower symptom severity 6 years later, and less reaction time variability predicted better overall functioning. Percentage of explained variance for the neurocognitive predictors was small (3.0–5.6 %) but significant. Together with baseline behavior, neurocognitive predictors remained significant, and a higher percentage of variance was explained compared to the percentage of variance explained by the baseline behavioral measures or neurocognitive measures alone.

The current finding that both better working memory and less reaction time variability contribute to better outcomes is in line with our hypothesis that better early neurocognitive functioning is associated with a positive outcome of ADHD. Interestingly, our finding is also consistent with the results of a recent study in which ADHD symptoms were assessed on a continuum both at baseline and follow-up, showing that better working memory and less reaction time variability predicted future ADHD symptoms and academic achievement, over and above baseline behavioral symptoms (Sjöwall et al. 2015). Notably, also other studies suggested that impairments in working memory and larger reaction time variability are most prominent of all neurocognitive functions involved in ADHD (Castellanos and Tannock 2002; Martinussen et al. 2005; Tamm et al. 2012). Previous studies, however, did not find predictive value of both verbal and spatial working memory for future ADHD status or symptoms (Biederman et al. 2009; Coghill et al. 2014). These discrepant findings may relate to differences between other studies and our study; e.g., smaller sample size (Biederman et al. 2009; Coghill et al. 2014), using dichotomous outcome measures compared to continuous measures (Biederman et al. 2009), and longer lengths of follow-up interval (Biederman et al. 2009). Also, previous studies measured the domain-specific aspects of phonological loop or visuo-spatial sketchpath (e.g., digit span or arithmetic subtests of the WISC/WAIS [Biederman et al. 2009] or visuospatial working memory in a forward form [Coghill et al. 2014]). In our study, we used a component measure of verbal (both a forward and backward condition) and visuospatial working memory. Possibly, with such a measure, we investigated a more domain-general central executive aspect of working memory (Kofler et al. 2014), which may have greater predictive power and may explain discrepancies in findings from other studies. By using different measures and tasks of working memory, we also tried to avoid a general issue regarding working memory, namely the debate about the exact underlying neurocognitive function that is assessed with span tasks (Aben et al. 2012; Cowan 2008; Davelaar 2013). Our finding that better working memory specifically predicted lower hyperactivity/impulsivity symptom severity, is in line with one study showing that the performance in the domain-general central executive aspect of working memory was significantly related to children’s activity level, which suggests that hyperactivity may act as a compensatory mechanism for working memory (Rapport et al. 2009). Regarding reaction time variability, to our knowledge, no other study investigated this neurocognitive function as predictor for future ADHD symptom severity or overall functioning.

Surprisingly, early inhibitory functioning and timing abilities did not predict current symptom severity or overall functioning. These results contrast the view that these functions act as core deficit in ADHD (Barkley 1997; Durston et al. 2011; Toplak et al. 2006). Furthermore, these results are inconsistent with previous studies in our sample showing large deficits within the domain of inhibitory functioning and timing (Rommelse et al. 2008a, b; c; Rommelse et al. 2008a, b; c; Rommelse et al. 2007a, b; c). However, earlier studies confirm that inhibitory functioning and timing abilities may not predict ADHD outcomes (McAuley et al. 2014; van Lieshout et al. 2013). Our data suggest that relations between neurocognitive functioning and ADHD outcomes over time may differ between neurocognitive functions that are thought to be closely related.

There are some alternative explanations that should be discussed in relation to our findings. The finding that many of our baseline neurocognitive measures did not predict future outcomes, gives rise to the thought that the development of neurocognitive performance may not relate to ADHD outcomes at all, which would theoretically be of interest. In addition, it is possible that neurocognitive functioning and overall functioning are not evenrelated to each other at baseline (such as ADHD and neurocognitive functioning), which would explain that we did not find a longitudinal relationship between most of our neurocognitive measures and overall functioning in particular.

In this study, we addressed questions regarding age, gender, pharmacological treatment, follow-up interval and study site, to contribute to existing literature. Previous studies revealed mainly negative findings on the predictive value of early neurocognitive functions for ADHD outcomes. As brain development is thought to be non-linear (Giedd et al. 1999), with, for example, a developmental spurt in adolescence (Casey et al. 2011), we expected moderating effects of age on the relation between relevant neurocognitive predictors and ADHD outcomes. Surprisingly, our models were independent of age. Similarly, predictive effects were independent of gender. According to our findings, relations between both working memory and response variability and ADHD outcomes were not confounded by the duration of medication taken until follow-up. Moreover the duration of medication taken had no beneficial effect on our ADHD outcomes, as medication use was a non-significant predictor in the models. This finding is consistent with findings of the MTA study showing that over 6 to 8 years, there was no advantage of pharmacological treatment on ADHD outcomes (Molina et al. 2009). Long-term benefits on academic and occupational outcomes, social functioning and comorbidities are also questionable (Langberg and Becker 2012; van de Loo-Neus et al. 2011). Pharmacological treatment is one of the preferred treatments in ADHD and may have adverse side effects as well. Our results and previous findings failing to support long term benefits of pharmacological treatment stress the importance of more work in this important area of research. Another issue is that follow-up interval was related to a lower K-GAS-score. Further exploring this relationship showed us that younger children were the ones with longer follow-up intervals; it may thus well be possible that this finding reflects the larger impact ADHD has on the overall functioning of younger children. Study site was not a factor of relevance to our data.

Taken together, the predictive value of neurocognitive functioning for ADHD outcomes is small; smaller than we expected based on the well-established and moderate to strong relationship between ADHD behavior and neurocognitive functioning observed cross-sectionally. The small relationship between baseline neurocognitive functioning and behavioral outcomes on the long term that we found, together with earlier findings on this topic (Coghill et al. 2014; McAuley et al. 2014; Rajendran et al. 2013a, b; van Lieshout et al. 2013) shows that neurocognitive functioning may not be seen as protective or a risk factor for longer term behavioral outcomes. These findings clearly indicate that further research is needed to understand the role or neurocognitive functioning in ADHD, for example by setting up longitudinal studies that look into more complex interactions of neurocognitive functioning with genetics and environment (e.g., family environment, peer group influences) and look at more than symptom outcome and functioning such as social behavior and self-esteem (Savitz et al. 2007). Based on our findings, it may be suggested that working memory and variability in responding are the promising neurocognitive measures that can contribute to such multimodal prediction models.

Clinically, our findings indicate that although working memory and variability in reaction time are independently predictive of ADHD outcomes, these effects are of such small magnitude that our findings are yet of little relevance for reliably establishing prognosis. This means that we are not able to predict which children with ADHD will improve on their level of behavioral symptoms and/or impairment based on their neurocognitive performance at an earlier time point. Our findings do not rule out the possibility that neurocognitive profiling to establish current strengths and weaknesses still may be of relevance for improving and supporting (school/occupational) functioning at the current moment, or to help understand and explain certain behavioral problems or impairments.

Some limitations should be noted. First, some aspects of our sample limit generalization to the population, including our exclusive focus on participants with the combined type of ADHD (Lara et al. 2009), the limited representation of girls in our sample and the inclusion of Caucasian individuals. Second, we did not verify medication use with the participant, which may have resulted in a less than optimal estimation of medication use in reality. Third, although we did include a broad array of neurocognitive functions, we were not able to include all neurocognitive domains currently regarded important in ADHD, such as reward related neurocognitive functions. Our findings thus cannot be generalized to other neurocognitive domains. Fourth, we have chosen to use performance-based measures of neurocognitive functioning. Rater-based measures of neurocognitive functioning may show higher predictive value, as these measures may be more closely related to behavior and investigate capacities in more unstructured situations, which may better mirror ‘real-life’ functioning. Fifth, for the investigation of the value of neurocognitive functioning over and above baseline behavior, we used the exact same measures for baseline symptom severity and follow-up symptom severity. This may partly explain the higher percentage of explained variance for baseline symptoms compared to neurocognitive functioning.

To further disentangle the complex relation between neurocognitive functioning and ADHD symptoms, future studies could take into account both neurocognitive functioning at baseline and at follow-up, in order to look at the relation between neurocognitive development over time and the course of ADHD. In addition, a person-based analysis in which neurocognitive profiles within one person are investigated might shed more light on this issue as well. Patterns of neurocognitive performance within one person might be better suited to predict future outcomes such as symptom severity and overall functioning. Such approaches might enable us to further understand the complexity of the development of behavior and understanding mechanisms of recovery from problematic behavior. Adding key concepts in ADHD such as reward processing may add to a more complete understanding of ADHD.

In conclusion, using a broad array of early neurocognitive functions to predict current ADHD symptom severity and overall functioning, we found only little evidence for the hypothesis that a stronger neurocognitive profile (better working memory, smaller reaction time variability) predicts better outcome. Our findings challenge the role of neurocognitive functioning in the long term outcome of ADHD.

## Electronic supplementary material

Table S1 Supplement (DOCX 18 kb)
