# Supplementary material for: Neurocognitive Predictors of ADHD Outcome: a 6-Year Follow-up Study
Source: J Abnorm Child Psychol. 2016 Jul 9;45(2):261–72. doi: 10.1007/s10802-016-0175-3 (PMC5241361; doi:10.1007/s10802-016-0175-3)
Supplement: Supplementary file 1 — Supplement (DOCX 18 kb) [file 10802_2016_175_MOESM1_ESM.docx]

Table 1 Supplement

*Correlations between baseline and follow-up behavioral measures and neurocognitive predictors*

|  |  | Pearson correlation (*r*) | | |  |  |  |  |  |  |  |  |  |
| --- | --- | --- | --- | --- | --- | --- | --- | --- | --- | --- | --- | --- | --- |
|  |  | 1 | 2 | 3 | 4 | 5 | 6 | 7 | 8 | 9 | 10 | 11 | 12 |
| 1 | Baseline ADHD symptom severity (CPRS-R:L scale N) | - |  |  |  |  |  |  |  |  |  |  |  |
| 2 | Follow-up ADHD symptom severity (CPRS-R:L scale N) | **0.32^**^** | - |  |  |  |  |  |  |  |  |  |  |
| 3 | Baseline impairment  (SDQ impairment parent) | **0.44^**^** | **0.36^**^** | - |  |  |  |  |  |  |  |  |  |
| 4 | Follow-up overall functioning (K-GAS-score) | -0.11 | **-0.22^**^** | **-0.18^**^** | - |  |  |  |  |  |  |  |  |
| 5 | PC working memory | -0.13 | **-0.19^**^** | -0.12 | **0.15^*^** | - |  |  |  |  |  |  |  |
| 6 | PC motor inhibition | -0.07 | -0.04 | 0.04 | 0.11 | **0.38^**^** | - |  |  |  |  |  |  |
| 7 | PC cognitive inhibition | -0.04 | -0.10 | -0.01 | 0.02 | **0.34^**^** | 0.07 | - |  |  |  |  |  |
| 8 | PC reaction time variability | -0.09 | -0.10 | **-0.15^*^** | **0.14^*^** | **0.55^**^** | **0.53^**^** | **0.18^**^** | - |  |  |  |  |
| 9 | PC timing | -0.11 | -0.07 | -0.07 | **0.14^*^** | **0.59^**^** | **0.43^**^** | **0.21^**^** | **0.70^**^** | - |  |  |  |
| 10 | PC information processing speed | -0.07 | -0.05 | -0.06 | 0.08 | **0.60^**^** | **0.37^**^** | **0.36^**^** | **0.64^**^** | **0.57^**^** | - |  |  |
| 11 | PC motor control | -0.07 | -0.07 | -0.07 | **0.15^*^** | **0.42^**^** | **0.37^**^** | **0.17^*^** | **0.49^**^** | **0.48^**^** | **0.56^**^** | - |  |
| 12 | Intelligence (TIQ) | -0.09 | -0.05 | -0.10 | 0.06 | **0.30^**^** | 0.04 | **0.17^*^** | **0.23^**^** | **0.24^**^** | **0.16^*^** | 0.03 | - |

* Correlation is significant at the 0.05 level (2-tailed). ** Correlation is significant at the 0.01 level (2-tailed). **ADHD** Attention-Deficit/Hyperactivity Disorder. **CPRS** Conners’ Parent Rating Scale-Revised: Long version. **K-GAS** Kiddie-Global Assessment Score. **SDQ** Strengths and Difficulties Questionnaire. **PC** Principal Component.
